# Addressing the critical need for open experimental databases in materials science

**DOI:** 10.1016/j.patter.2021.100411

**Published:** 2021-12-10

**Authors:** Matthew K. Horton, Rachel Woods-Robinson

**Affiliations:** 1 Materials Sciences Division, Lawrence Berkeley National Laboratory, Berkeley, CA, USA; 2 Department of Materials Science and Engineering, University of California, Berkeley, Berkeley, CA, USA; 3 Applied Science and Technology Graduate Group, University of California, Berkeley, Berkeley, CA, USA

## Abstract

With the HTEM, an open online database containing experimental synthesis and characterization data of thin film inorganic materials, Talley et al. (2021) lay a foundation for a new era of high-throughput materials design.

## Main text

The age of big data is transforming the way that science is performed, with materials discovery benefiting from the accessibility of large datasets. Aggregating results from multiple researchers can allow unique insights to be uncovered, and having datasets available in machine-readable formats can also unlock their potential for use in combined experimental-computational studies. However, to date there have been relatively few open experimental property databases in the materials sciences. While some pioneering experimental databases do exist that are now quite large or extensive, such as Pauling File^1^ and others, they are usually locked behind commercial licenses or have restrictions placed on their use. Data formats are also an obstacle, with some data existing only graphically or in proprietary formats designed by equipment manufacturers. Furthermore, while databases of properties do exist, they typically do not make the underlying instrument measurement available, leading to issues in reproducibility or verification of methods.

These data-related challenges arise not only from the wide range of accessible material space—various compositions, polymorphs, off-stoichiometries, microstructures, etc.—but also from the new methods of growth and characterization increasingly available to researchers. These include “high-throughput” synthesis techniques, such as combinatorial growth,^2^ which can now generate “libraries” of phases across vast swaths of composition space as well as characterization methods that generate large, multi-dimensional data such as modern synchrotron and microscopy techniques. Such datasets simply cannot be adequately expressed in a paper figure and require alternative interfaces to fully represent.

**Figure 1:**
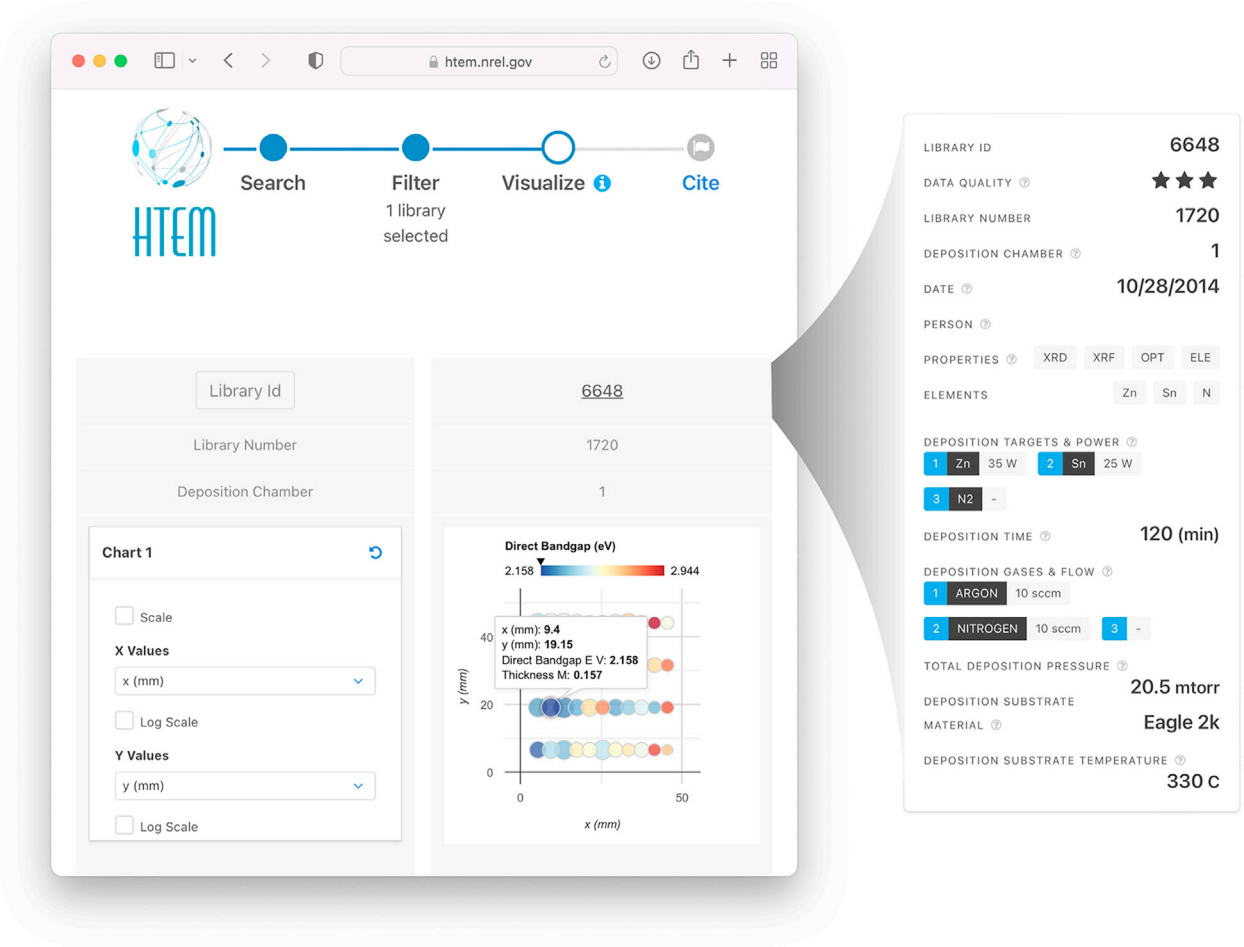
An example visualization of a combinatorial growth library on the HTEM database's website The graph of colored circles on the right represents the “library,” a thin-film material grown across a gradient of deposition conditions (usually a composition and temperature gradient). The library is subdivided into samples, in which the circles represent a given x-y position, and each sample contains experimental property measurements (in this case bandgap and thickness). The inset on the right shows metadata about the library such as deposition details and is also included on the website.

In this issue of *Patterns*, Talley et al.^3^ have detailed the framework behind their High-Throughput Experimental Materials (HTEM) database,^4^ which addresses some of these critical community challenges. The HTEM contains data collected during combinatorial experiments at the United States’ National Renewable Energy Laboratory (NREL) over the past 10 years, containing over 1,800 libraries of inorganic thin-film materials and associated characterization data. Each thin-film library typically contains 44 data points per library and is grown across a gradient of temperature and composition (see Figure 1 for an example library). These libraries and properties are presented in a usable web app for examining and comparing individual data entries and also, critically, with an open API that can return the same data in a machine-readable format. Examples of using this API with the Python programming language to retrieve data are also made available by the authors.

Perhaps most notable about this work is how the HTEM database has seen a large number of regular contributions. Historically the adoption of many technical advances has been fundamentally limited by system design, specifically by a lack of design that considers the human being using the system. Design is not just a veneer on top of a technical system; it is often the reason that a system succeeds or fails in the long term. In this sense, making a database system that works is only half the problem; ensuring its continued use by the community it serves is the other half. At the time of publication of this paper, this database has seen continued contributions from over 30 researchers, which is a significant testament to its adoption within the NREL community and bodes well for its future scalability. This usage and scalability is enabled by the robust infrastructure described in this work and is an example of how to design a data platform that has to integrate with a variety of experimental instruments and handle a large quantity of hetereogenous data.

As we look back on decades of publishing in the materials sciences, particularly since the start of electronic publishing, it has been difficult to promote data accessibility, leading to large quantities of data locked inside tables, figures, dor supporting information. There are even current machine learning efforts to parse information from text and static images that could have been avoided had the data been shared appropriately at the start. However, it is not sufficient that data be made available in a machine-readable format; it must also be available under an open license that allows the reuse necessary for scientific advancement. To this end, the authors have announced their public datasets are available under a Creative Commons license.^5^ This type of license is particularly appropriate for scientific work since the underlying research is typically performed using public funds, so it is essential that resulting data outputs are also available for public use. Encouragingly, use of these licenses is now becoming more commonplace, especially with the recent promotion and adoption of FAIR principles by the broader community. PuRe data resources are another example of the increased focus on encouraging reuse of data stored in public databases.

Storing and retrieving data in a standardized manner is also helpful for the individual researcher, who might use this system to track the provenance of their own data. Experimentalists tend to create their own *ad hoc* data structures, which can be time intensive, inconsistent across researchers, and thus may be difficult to share with the community. Instead, the HTEM database offers a coherent platform to “collect, process, and store experimental data and metadata” consistently and systematically. Furthermore, it allows both experimental and computational researchers to easily query previous experiments. This can reduce duplicate effort, prevent accidental data mismanagement that can lead to erroneous results or even data loss, and, importantly, help inform future research endeavors.

New experimental databases of this kind complement the open computational databases, such as the Materials Project, the Open Quantum Chemistry Database, and other efforts inspired by the Materials Genome Initiative, that are already in existence. While these computational databases have proved very popular and valuable, their utility is fundamentally contingent on the ability to validate predictions with comparison to experiment.^6^ The Materials Project has made some inroads here by allowing experimental data to be uploaded alongside computational predictions through its user contribution platform (“MPContribs”), but this is only a start and a cross-validation component remains to be developed. A future whereby computational and experimental databases are able to link between and support each other to their mutual benefit is the next grand challenge in our community. Efforts such as the HTEM database will be crucial for establishing a rigorous foundation for the next era of data-driven materials science and promise a future where design and discovery of materials for urgent applications, such as those presented by our current climate crisis, might be greatly accelerated.

## Web resources

FAIR Principles, https://www.go-fair.org/fair-principles/

High-Throughput Experimental Materials Database, https://htem.nrel.gov/

Materials Project, https://materialsproject.org/

Open Quantum Chemistry Database, http://oqmd.org

PuRe Data Resources, https://science.osti.gov/Initiatives/PuRe-Data/Resources-at-a-Glance
